# Galectin-3 induced by hypoxia promotes cell migration in thyroid cancer cells

**DOI:** 10.18632/oncotarget.21135

**Published:** 2017-09-21

**Authors:** Jiaojiao Zheng, Weihui Lu, Cong Wang, Yang Xing, Xiaoning Chen, Zhilong Ai

**Affiliations:** ^1^ Zhongshan Hospital of Fudan University, Shanghai, People's Republic of China; ^2^ Key Laboratory of Glycoconjugates Research, Ministry of Public Health, Department of Biochemistry and Molecular Biology, Shanghai Medical College of Fudan University, Shanghai, People's Republic of China

**Keywords:** galectin-3, thyroid cancer, hypoxia, migration, Src signaling pathway

## Abstract

**Background:**

The aim of this study is to investigate the role of Galectin-3 in human thyroid cancer migration.

**Methods:**

The expression of Galectin-3 in surgical specimens was investigated using immunohistochemistry and western blot. A papillary thyroid cancer cell line (B-cpap) and an anaplastic thyroid cancer cell line (8305c) were transfected with short-hairpin RNA against Galectin-3 (Gal-3-shRNA). Low-molecular citrus pectin (LCP) was also used to antagonize Galectin-3. The migration and invasion of the cell lines were examined. The related signaling pathways were investigated to explore the Galectin-3 mechanism of action.

**Results:**

Galectin-3 was highly expressed in metastasized thyroid cancers. Knocking down and antagonizing Galectin-3 significantly suppressed the migration of thyroid cancer cells. Knocking down Galectin-3 inhibited the activity of Wnt, MAPK, Src and Rho signaling pathways. Galectin-3 was up-regulated via HIF-1α in a hypoxic environment. Galectin-3 knockdown could reduce cell motility in hypoxic environments.

**Conclusion:**

This study suggests that Galectin-3 could act as a modulator of thyroid cancer migration, especially in hypoxic microenvironments. This regulation function of Galectin-3 may work through multiple signaling pathways.

## INTRODUCTION

Thyroid cancer is one of the most common carcinomas in human beings, with its incidence growing rapidly over the last 20 years. Among the emerging cases, most are papillary cancers [1]. Papillary thyroid cancer (shorted as PTC) is a low malignant cancer with its twenty-year survival rate over 90% [2]. Despite its good prognosis, some subtypes of PTC also have aggressive properties, such as early local invasion or extensive lymph node metastasis [3]. The mechanisms of how PTC metastasizes are still not well understood. In contrast, anaplastic thyroid cancer (shorted as ATC) is a rare type of thyroid cancer with a generally poor prognosis. Most patients with ATC survive for only 4 to 12 months after diagnosis [4]. Studies have revealed that some types of ATC may be the long-term consequence of PTC dedifferentiation [5, 6]. The relationship between PTC and ATC is worth studying because it may help to understand the biological behavior of both diseases and further improve their clinical treatments.

Galectins are a family of carbohydrate-binding proteins which have high affinity and specificity for β-galactoside [7]. Among Galectins, Galectin-3 is one of the best studied, which mediates multiple processes of tumor growth [8]. Galectin-3 has been proven to be associated with the metastasis and invasion of various cancers. In tongue cancer cells, Galectin-3 mediates tumor metastasis and invasion through Wnt/β-catenin signaling pathway and Akt phosphorylation [9]. Similar results have been obtained in pancreatic cancers [10]. Galectin-3 increases the expression of MMP1 in gastric cancer through the activation of PAR-1 signaling, thus causing metastasis [11]. Circulating Galectin-3-MUC1 accounts for cancer cell embolus formation and the survival of circulating cancer cells in colon and breast cancers [12].

For thyroid cancers, detection of Galectin-3 expression has been widely used in Fine Needle Aspiration Cytology (FNAC) to help distinguish benign and malignant thyroid nodules [13, 14]. Overexpression of Galectin-3 in human thyroid follicular cells enhanced malignant transformation [15]. Galectin-3 in association with K-Ras GTP contributes to thyroid carcinoma malignancy in anaplastic thyroid cancer cells [16]. Although many studies have been conducted on the mechanism responsible for Galectin-3 modulation of cancer progressions, its role in cancer metastasis of thyroid is still not well understood.

In this study, we used surgical specimens and two different thyroid cancer cell lines to investigate how Galectin-3 was associated with thyroid cancer cell migration. The potential signaling pathways through which Galectin-3 regulating migration were also explored. Our results mainly revealed that Galectin-3 regulated thyroid cancer cell migration in normoxic and hypoxic microenvironments. The signaling pathways involved included Wnt, MAPK, Src and Rho.

## RESULTS

### Galectin-3 was highly expressed in PTC tissues and metastasized lymph nodes

**Figure 1:**
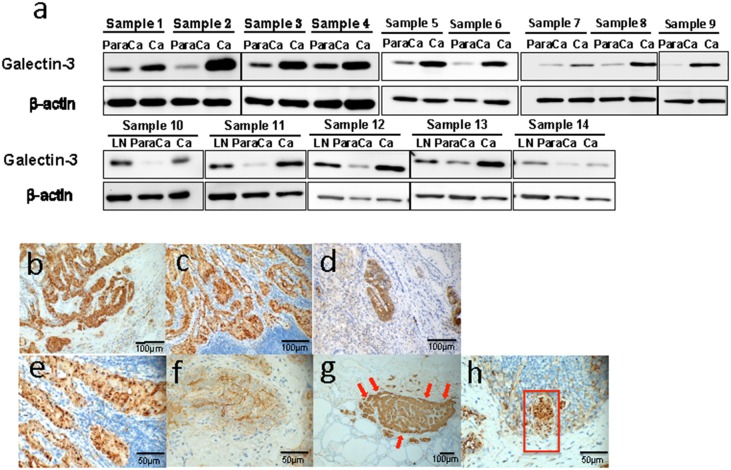
Galectin-3 was highly expressed in thyroid cancers and was related to cancer migration **(a)** Western blot analyses of Galectin-3 expression in peritumor tissues (para Ca), thyroid carcinomas (Ca) and metastasized lymph nodes (LN). The definition of peritumor tissues in our study is thyroid tissues 2-5 mm adjacent to the cancer site. (b-h) Immunohistochemistry analyses of Galectin-3 expression in thyroid cancer samples. **(b-c)** Representative photographs of Galectin-3 expression in papillary thyroid cancer (Magnification 200 X) and the metastasized lymph node (Magnification 200 X) from the same patient. **(d)** Representative picture of Galectin-3 expression in anaplastic thyroid cancer (Magnification 200 X). **(e-f)** Different distribution of Galectin-3 in nucleus (e) (Magnification 400 X) and intercellular spaces (f) (Magnification 400 X) in papillary thyroid cancers. **(g)** Representative photograph of Galectin-3 expression in papillary thyroid cancer, which had comparatively stronger staining at the frontier of the tumor mass (Magnification 100 X). **(h)** Identification of a mass of tumor cells (within the red frame) with half of it highly expressing Galectin-3 (Magnification 400 X).

To determine the expression characteristics of Galectin-3 in different thyroid tissues, the protein levels of Galectin-3 in surgical specimens from 14 papillary thyroid cancer patients were analyzed by western blot. 5 of the 14 patients were diagnosed with metastasized lymph nodes. Galectin-3 was highly expressed in papillary tumor tissues compared to its adjacent noncancerous thyroid tissues. Furthermore, all metastases in lymph nodes also showed high Galectin-3 expression (Figure 1a).

To further confirm the expression patterns of Galectin-3 in thyroid cancer, we conducted immunohistochemistry in 20 surgical specimens, including 19 pathology-confirmed papillary thyroid cancer samples and 1 anaplastic cancer sample. The related information is listed in Table 1. Most papillary thyroid cancers showed strong or at least medium Galectin-3 expression in both cancers and metastases (Figure 1b&1c). While the anaplastic cancer tissue showed an extensive but weak Galectin-3 expression level. Only some spots in the very middle of it exhibited strong Galectin-3 expression (Figure 1d). These spots were papillary-structured, implying the possible relationship between papillary thyroid cancer and anaplastic thyroid cancer. There was a significant difference on immunohistochemistry between papillary cancer patients with lymph node metastases and patients without metastases (4.0 ± 1.04 Vs. 2.2 ± 1.09, *p* < 0.05). Through investigating these slides carefully, different distributions of Galectin-3 could be defined. On Figure 1e, Galectin-3 was mainly located in nucleus and cytoplasm while on Figure 1f, Galectin-3 was detected in cytoplasm and intercellular spaces. Another phenomenon that was found in some specimens (2/19) was the higher expression of Galectin-3 in the tumor frontier (Figure 1g). Figure 1h showed a cluster of PTC cells invading a minute lymph node. Half of this cluster was strongly positive for Galectin-3 and this half was also the head of this invading metastasis. Thus, the expression of Galectin-3 was high in PTC tissues and metastasized lymph nodes.

**Table 1:** Relationship between the expression of Galectin-3 and histopathologic features of papillary thyroid cancers

Case no.	Age	Gender	Metastasis in lymph nodes	Tumor stage	Galectiin-3 score
1	58	F	Y	IV	2
2	38	M	Y	I	3
3	29	F	Y	I	4
4	53	F	Y	IV	4
5	40	M	Y	I	5
6	32	F	Y	I	4
7	55	F	Y	IV	4
8	26	F	Y	I	5
9	40	F	Y	I	5
10	25	F	Y	I	2
11	42	F	Y	I	5
12	36	F	Y	I	5
13	25	F	N	I	2
14	43	F	N	I	2
15	26	F	N	I	2
16	61	M	N	I	1
17	30	F	Y	I	4
18	47	F	N	I	4
19	38	M	Y	I	4
20(ATC)	62	M	Y	IV	1

^*^Immunohistochemical (IHC) staining was estimated as follows: **0:** no signal; **1:** signal in ≤10% of cells; **2:** signal in 10%-25% of cells; **3:** signal in 25%-50% of cells; **4:** signal in 50%-75% of cells; **5:** signal in >75% of cells. Y=metastasis N=no metastasis.

### Inhibition of Galectin-3 decreased the invasion and migration of thyroid cancer cells

**Figure 2:**
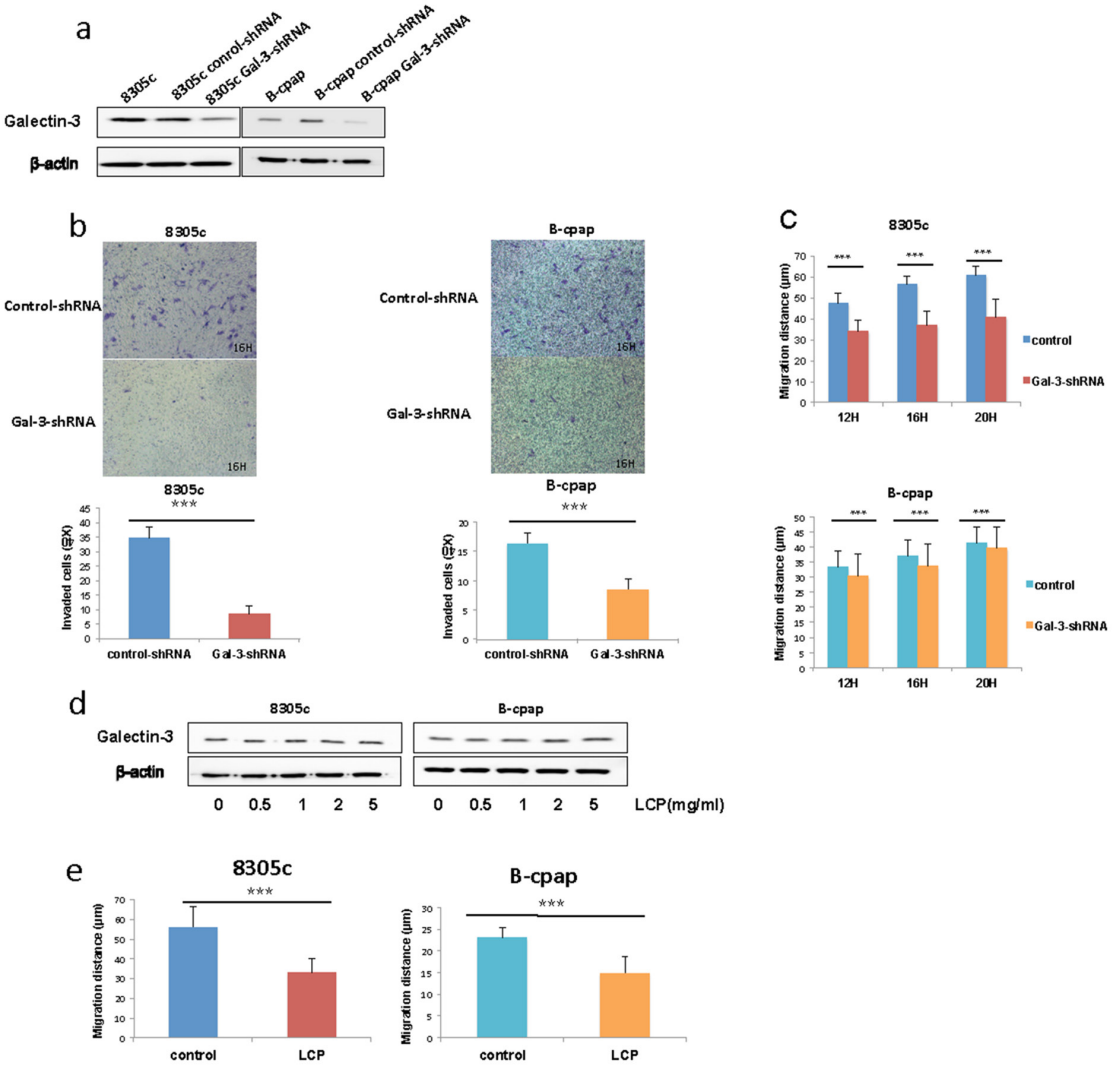
Inhibition of Galectin-3 suppressed thyroid cancer cell migration and invasion **(a)** Western blot analysis of Galectin-3 expression in the B-cpap cell line and 8305c cell line after transfection with control- or Gal-3-shRNA lentivirus. **(b-c)** Transwell assays and wound healing assays conducted in 8305c and B-cpap cell lines respectively after transfection. Statistical analysis was performed using Student's t test (^*^, *p* < 0.05; ^***^, *p* < 0.001; ns, not significant). **(d)** Western blot analysis of Galectin-3 expression in the B-cpap cell line and 8305c cell line with inhibition of Galectin-3 by LCP of different concentration. **(e)** Wound healing assays conducted in 8305c and B-cpap cell lines respectively with inhibition of Galectin-3 by 2 mg/ml LCP.

Based on our findings in immunohistochemistry and western blots of surgical samples, we first supposed that Galectin-3 played a role in the migration and invasion of thyroid cancers. Papillary thyroid cancer cell line B-cpap and anaplastic thyroid cancer cell line 8305c were chosen to knock down Galectin-3 (Figure 2a). Wound healing assays and Transwell assays were then performed to examine the role of Galectin-3 in thyroid cancer cell migration. The Transwell assays showed that Galectin-3 knockdown significantly decreased cell invasion (Figure 2b). Consistent with this, the migration distance of Galectin-3 knockdown cells were shorter than their control groups (Figure 2c). Since many of the functions of Galectin-3 rely on its binding partners [17], Galectin-3 was also inhibited with another method. We use LCP (low-molecular citrus pectin) to competitively block the interaction between Galectin-3 and its partners. Figure 2d showed the unchanged levels of intracellular Galectin-3 no matter which concentration of LCP was applied. Taking the cell tolerance into account, we applied a concentration of 2 mg/ml LCP to inhibit Galectin-3 function and performed wound healing assays. The migration distances of both 8305c cells and B-cpap cells were decreased (Figure 2e). Altogether, knocking down Galectin-3 or blocking its function attenuated the invasion and migration of thyroid cancer cells.

### Galectin-3 knockdown slightly reduced tumor cell proliferation in the B-cpap cell line and reduced sphere-formation in the 8305c cell line

**Figure 3:**
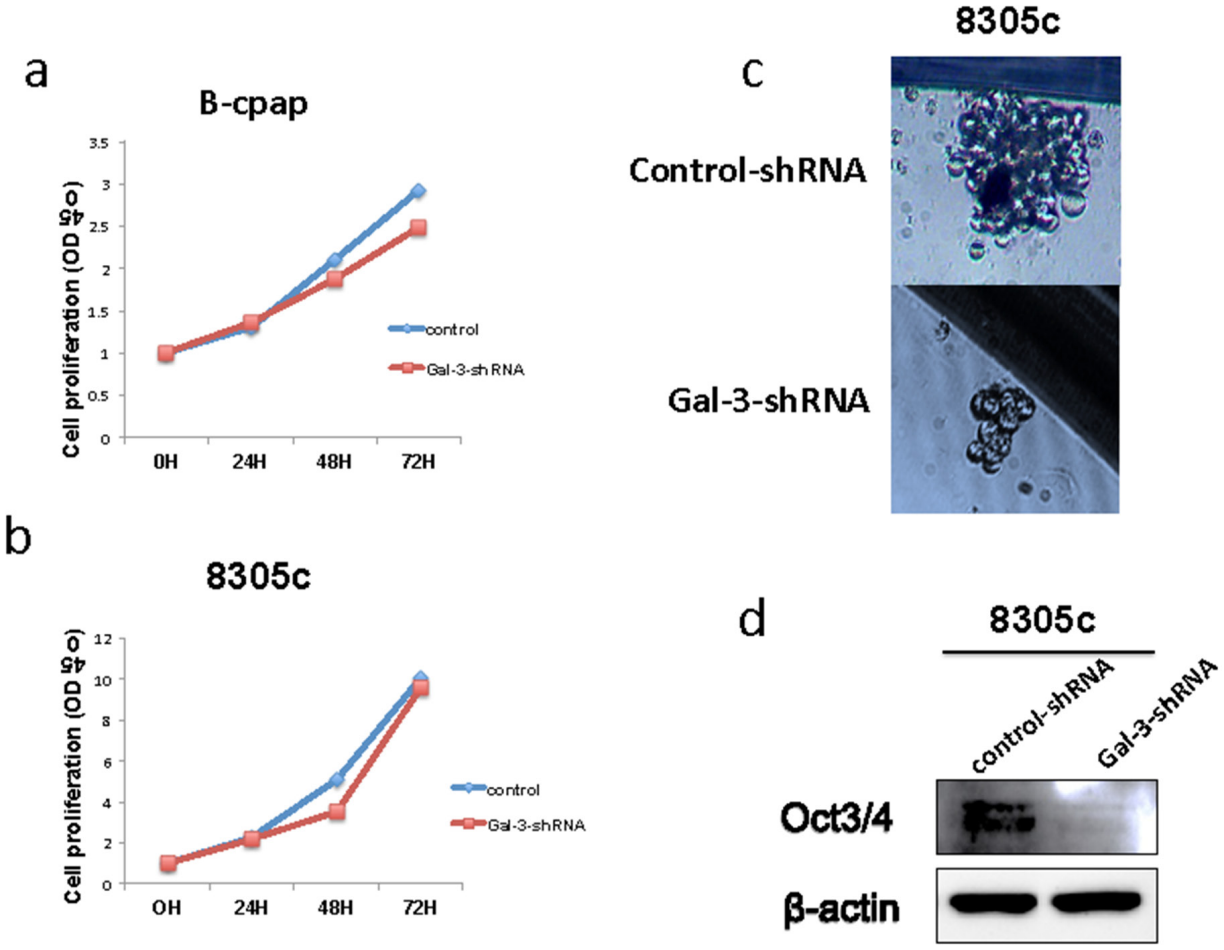
Down-regulation of Galectin-3 had different impact on thyroid cancer cell proliferation and their property of stem cell **(a-b)** Quantification of CCK8 assays in 8305c and B-cpap cells transfected with control- or Gal-3-shRNA. **(c)** Representative photographs of sphere formations of the 8305c cells after transfection. Photographs were taken after 14 days. **(d)** Western blot analysis of Oct3/4 expression in the 8305c cells after transfected with control- or Gal-3-shRNA.

Next, we performed CCK8 assays to examine the effect of Galectin-3 on thyroid cancer cell proliferation. Compared to control groups, B-cpap cells transfected with Gal-3-shRNA showed decreased cell proliferation (Figure 3a), while no differences were found between 8305c cells transfected with control- or Gal-3-shRNA (Figure 3b). A number of studies have indicated that tumor growth and proliferation is dependent on a small subset of cells, defined as cancer stem cells [18]. In order to illuminate the effect of Galectin-3 on cancer stem cell properties of thyroid cancers, we performed sphere formation assays in both cell lines. B-cpap cell line failed to form spheres in stem cell culture media after 14 days. However, 8305c cell line did form spheres after 14 days and Galectin-3 knockdown cells ended with fewer and smaller spheres compared to control cells (average spheres per vision: 1.4 vs 0.9, *p* < 0.05; average diameter (μm) per sphere: 32.6 vs 25.7, *p* < 0.05) (Figure 3c). Accordingly, a decreased level of stem cell marker Oct3/4 was also found in 8305c cells transfected with Gal-3-shRNA (Figure 3d). Altogether, knocking down Galectin-3 slightly decreased tumor cell proliferation of B-cpap cells. While sphere formation of 8305c cells was inhibited after Galectin-3 down-regulation.

### Galectin-3 knockdown attenuated the activity of MAPK, Wnt/β-catenin, Src and Rho signaling pathways

**Figure 4:**
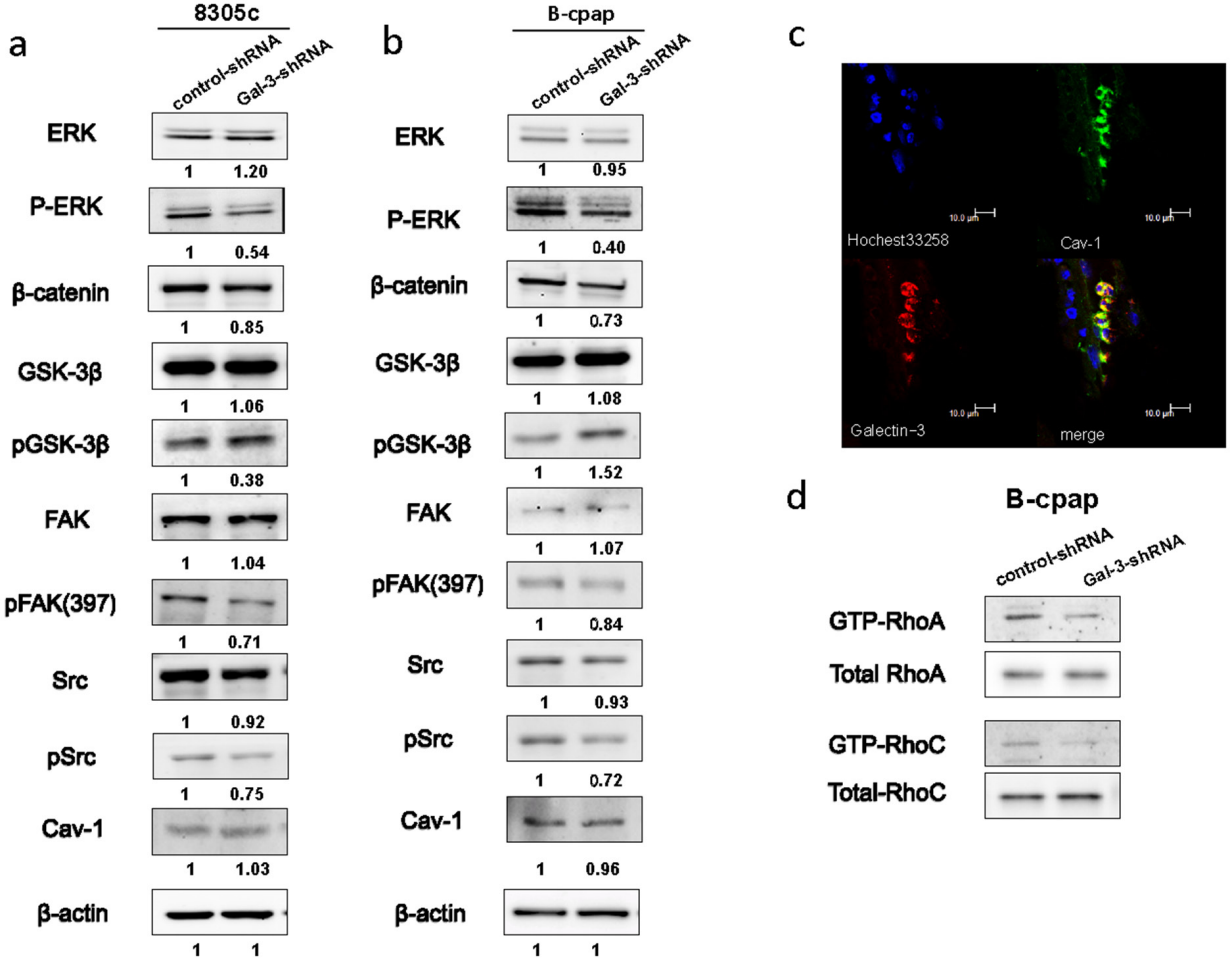
Down-regulation of Galectin-3 inhibited the phosphorylation of ERK, Src, and FAK, promoted the phosphorylation of GSK-3β, suppressed the expression of β-catenin and inhabited the activation of RhoA and RhoC **(a-b)** Western blot analyses of the expression levels of ERK/pERK, β-catenin, GSK-3β/pGSK-3β, FAK/pFAK, Src/pSrc and Cav-1 in both cell lines transfected with control- or Gal-3-shRNA. The quantification of the bands’ density was labeled behind. **(c)** Immunofluorescence assay was done to observe the co-localization of Cav-1 (green) and Galectin-3 (red). **(d)** RhoA GTPase and RhoC GTPase was assessed by pull-down of the GTP-Rho in B-cpap control cells and Galectin-3 knockdown cells.

To explore the mechanisms of Galectin-3 regulating the migration and invasion of thyroid cancer cells, we further investigated the effect of Galectin-3 knockdown on several signaling pathways related to cell migration. Since MAPK/ERK signaling is the most commonly studied signaling pathway in thyroid cancers [19], we examined the levels of ERK and phosphorylated ERK between thyroid cancer cells transfected with control- or Gal-3-shRNA. In both cell lines, Galectin-3 knockdown decreased the levels of phosphorylated ERK (p-44/42 MAPK). Since β-catenin is one of the binding partners of Galectin-3 and GSK-3β is the one of the binding partners of β-catenin [20], we also examined the expression levels of them and found out that β-catenin was suppressed and phosphorylated GSK-3β increased due to Galectin-3 knockdown (Figure 4a and 4b).

It has been proven that Src kinase-dependent caveolin-1 (Cav1) phosphorylation increased tumor cell migration in a Galectin-3-dependent manner [21]. The effect of Galectin-3 knockdown on expression levels of Cav-1, Src and its substrate FAK were also analyzed. A downward expression tendency of phosphorylated Src and phosphorylated FAK in Gal-3-shRNA transfected cells was disclosed compared to their control groups (Figure 4a and 4b). Although there existed no changes in the protein levels of Cav-1 between control cells or Galectin-3 knockdown cells, a co-localization of Galectin-3 (red) and Cav-1 (green) was found in papillary thyroid carcinoma tissue (Figure 4c). Rho GTPase activity was reported to be decreased after Galectin-3 silencing in hepatocellular carcinoma cells [22]. We also conducted pull down assays in B-cpap cells and detected the active forms of RhoA and RhoC. In B-cpap cells transfected with Gal-3-shRNA, levels of GTP-RhoA and GTP-RhoC were decreased (Figure 4d). Altogether, Galectin-3 knockdown led to the deactivation of MAPK, Wnt, Src and Rho signaling pathways.

### Galectin-3 was up-regulated by hypoxia

**Figure 5:**
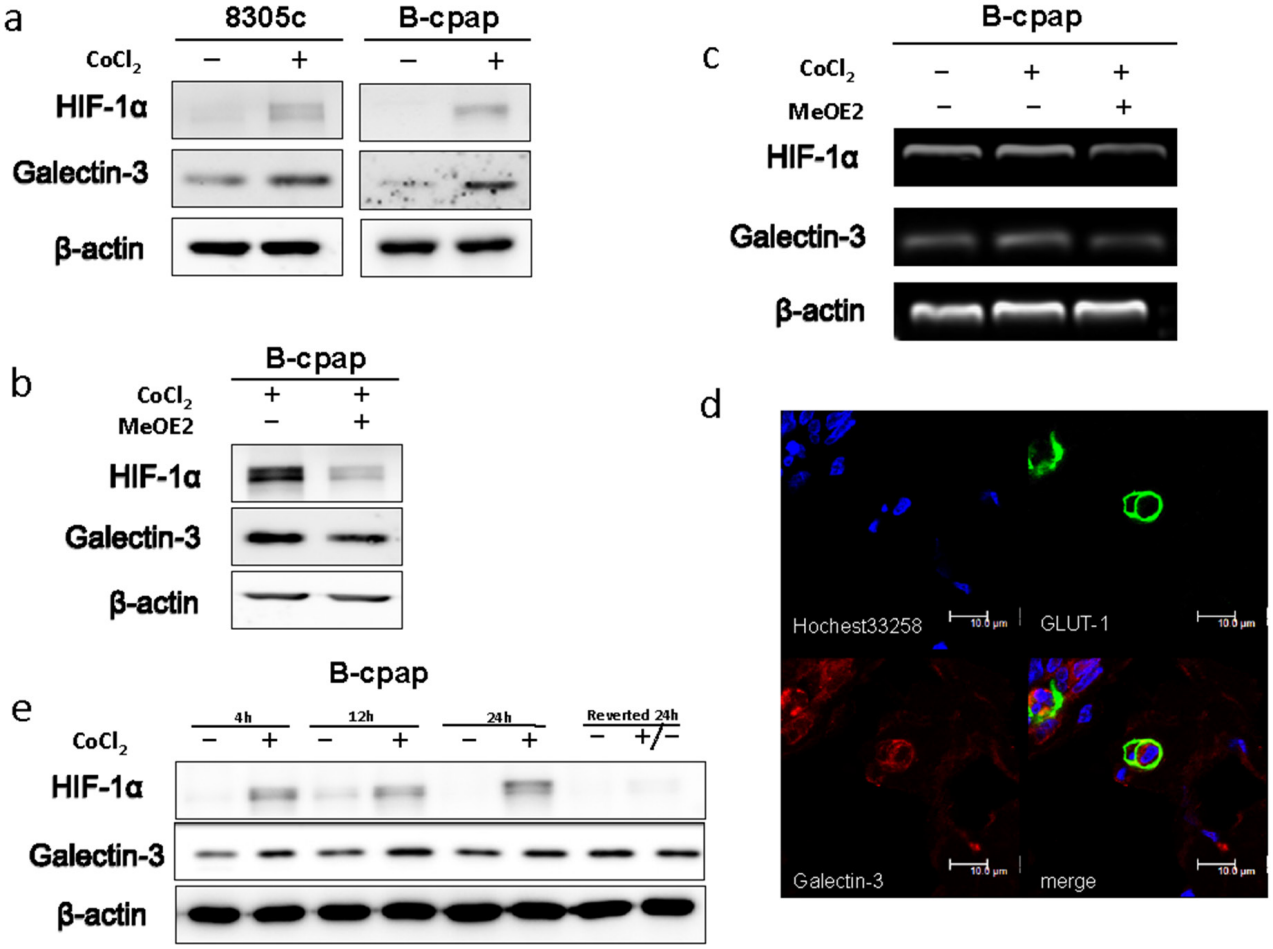
Hypoxia promoted the expression of Galectin-3 via a HIF-1α-dependent way **(a)** Western blot analysis of Galectin-3 and HIF-1αexpression in 8305c and B-cpap cells. Both of the cell lines were cultured in a complete medium or a medium with 100 μM CoCl_2_ for 36 hours. **(b)** Western blot analysis of Galectin-3 and HIF-1α expression in B-cpap cells treated with 100 μM CoCl_2_ or CoCl_2_ plus MeOE2 (10 μM) for 20 hours. **(c)** RT-PCR analysis of mRNA levels of LGALS3, HIF-1α and β-actin in B-cpap cells cultured in serum-free medium, serum-free medium with 100 μM CoCl_2_ and serum-free medium with 100 μM CoCl_2_ plus 10 μM MeOE2. **(d)** Immunofluorescence assay was done to observe the co-localization of Glut1 (green) and Galectin-3 (red). **(e)** Western blot analyses of HIF-1α and Galectin-3 expression in B-cpap cells with different periods of CoCl_2_ treatments.

All solid tumors would undergo a stress of lacking nutrient after growing to a certain size, which leads to a hypoxic microenvironment [23]. To imitate such environment, we added CoCl_2_ (100μM) to the culture medium. After 36 hours, Galectin-3 expression was markedly increased (Figure 5a). Hypoxia-inducible factor-1alpha (shorted as HIF-1α) is a transcription factor that commonly responds to the decreased oxygen levels in cellular environments [24]. Recent studies have shown that the expression of Galectin-3 was modulated by HIF-1α [25]. Inhibition of HIF-1α with MeOE2 (10μM) caused a significant decrease in Galectin-3 expression in our study (Figure 5b). An RT-PCR analysis was also performed to confirm the regulatory function of HIF-1α on Galectin-3 in mRNA levels (Figure 5c). Glucose transporter 1 (GLUT1), a gene directly regulated by HIF-1α, and Galectin-3 were double stained to confirm the coexistence of hypoxia and Galectin-3 expression in the surgical specimen (Figure 5d). To analyze whether Galectin-3 up-regulation in hypoxia would change with time, we further induced hypoxia of different time periods and investigated Galectin-3 expression levels in B-cpap cells. As shown in Figure 5e, Galectin-3 was up-regulated after B-cpap was cultured in hypoxia for 4 hours and remained unchanged till 24 hours. After the hypoxia was reverted for 24 hours, the expression of Galectin-3 decreased to normal level. Altogether, Galecitn-3 was up regulated by hypoxia in an HIF-1α dependent way.

### Galectin-3 knockdown inhibited CoCl_2_-induced cell migration

**Figure 6:**
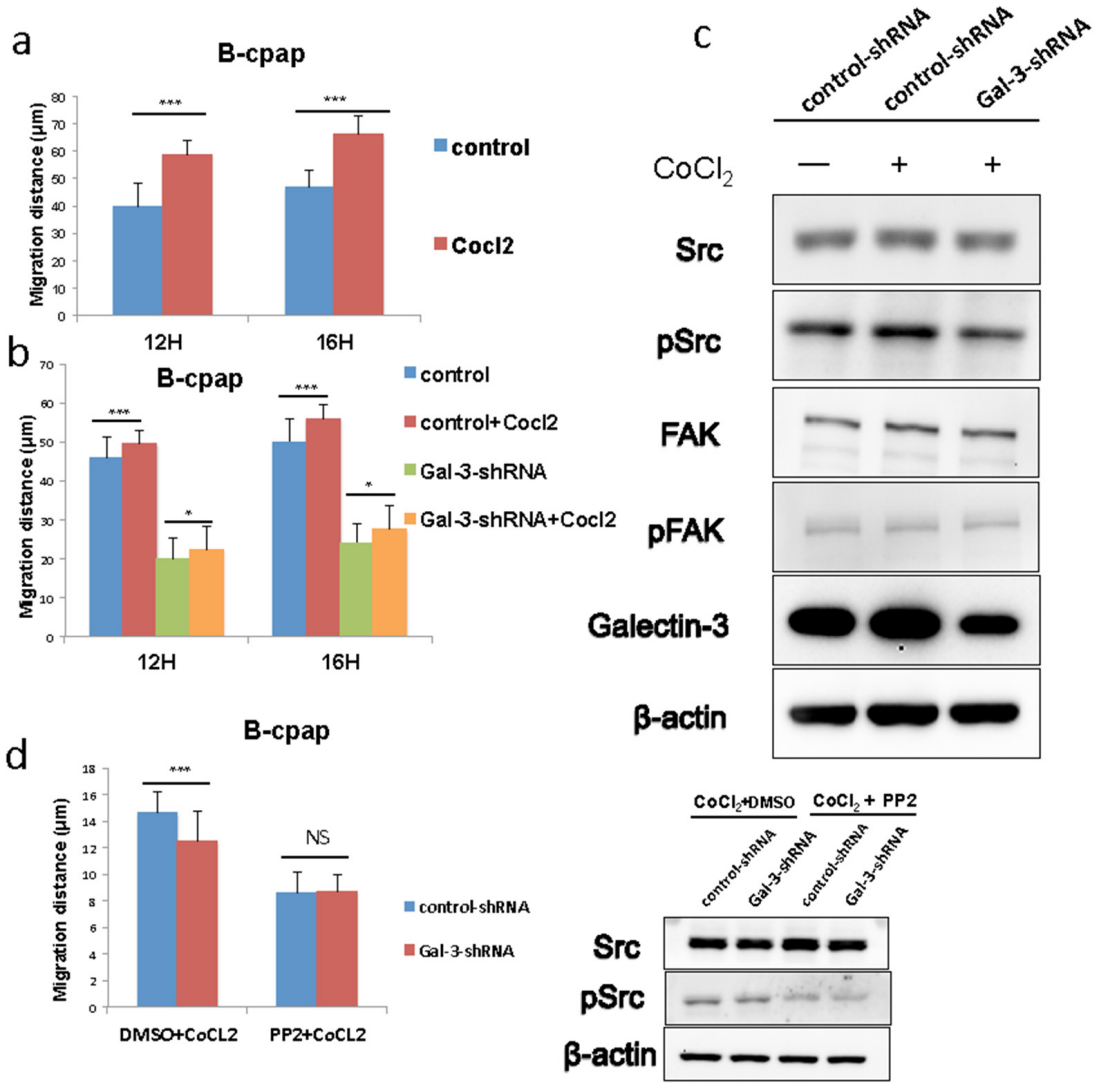
Galectin-3 was responsible for the increased migration of B-cpap cells in hypoxic microenvironment, and down-regulation of Galectin-3 offset part of this increase and induced Src deactivation **(a)** Quantifications of wound healing assays after 12 and 16 hours in B-cpap cells in normoxia or hypoxia. The hypoxic microenvironments were induced by 100 μM CoCl_2_. Statistical analysis was performed by Student's t test (^*^, *p* < 0.05; ^***^, *p* < 0.001; ns, not significant) **(b)** Quantifications of wound healing assays after 12 and 16 hours in B-cpap cells transfected with control- or Gal-3-shRNA in normoxia or hypoxia respectively. The hypoxic microenvironments were induced by 100 μM CoCl_2_. (^*^, *p* < 0.05; ^***^, *p* < 0.001; ns, not significant) **(c)** Western blot analysis of the expression of Src/pSrc, FAK/pFAK in B-cpap cells after transfection and CoCl_2_ treatment. The cells from left to right were control cells in nomoxia, control cells in hypoxia, Galectin-3 knockdown cells in hypoxia respectively. The control groups and treatment groups were harvested after 36 hours. **(d)** Quantifications of wound healing assays of B-cpap cells transfected with control- or Gal-3-shRNA in hypoxia or hypoxia with Src inhibitor PP2. The inhibitory effect of Src phosphorylation by PP2 was validated by western blot analysis. All of the cells were cultured in serum-free medium with 100 μM CoCl_2_ for 20 hours. DMSO was used as vehicle control.

In our previous studies, we had come to a conclusion that Galectin-3 was related to the migration of thyroid cancer cells. After the observation of the up-regulated Galectin-3 in hypoxic environments, we further assumed that this increase might bring out adaptive changes in cell migration. Papillary cancer cell line B-cpap was used to conduct wound healing assays in hypoxic and normoxic microenvironments. As a result, B-cpap cells in hypoxic groups migrated a longer average distance after 12 and 16 hours compared to those in normoxic groups (Figure 6a). Then the wound healing assays were repeated in B-cpap cells transfected with control- or Gal-3-shRNA. It turned out that in hypoxic environments, either control- or Gal-3-shRNA transfected cells migrated faster than their control groups, which were in normoxic environments. But differences between Galectin-3 knockdown groups were much slighter (Figure 5d, migration gap (μm): 12H 3.46 vs 2.3; 16H 5.8 vs 3.5, Figure 6b). In western blot assays, cells transfected with Gal-3-shRNA showed a decreased expression of phosphorylated Src compared to control cells. But Galectin-3 knockdown seemed to have no impact on the expression level of phosphorylated FAK in hypoxic environments (Figure 6c). In order to validate this result, Src inhibitor PP2 was added during wound healing assays. The results indicated that when Src signaling pathway was inhibited, Galectin-3 knockdown in hypoxic microenvironment did not significantly change the migration of B-cpap cells (Figure 6d). The inhibition effect of PP2 was validated by western blot. To conclude, the migration ability of B-cpap cells increased in hypoxia and knocking down Galectin-3 could partly offset this increase. The underlying mechanism mainly lay on the Src signaling pathway.

## DISCUSSION

Galectin-3 plays different roles in the process of tumor growth including cell differentiation [26], adhesion [27], migration [11], invasion [15], metastasis [28] and angiogenesis [29]. In this study, we explored the expression patterns of Galectin-3 in surgical specimens of thyroid cancers, investigated the effect of Galectin-3 inhibition on cell migration and tried to clarify its underlying signaling changes.

Thyroid cancers with Galectin-3 expression were prone to metastasize in some studies [30, 31]. In our study, high expression of Galectin-3 was also confirmed in PTC metastases. Metastasis is the spread of cancer cells from its primary sites to distant organs following new formation of tumors of the same type. This progression contains a series of complex steps including detachment, intravasation, escape from immune surveillance, extravasation, adhesion, proliferation and angiogenesis. The accomplishment of each these step is the premise of tumor metastasis [32]. The motility of cancer cells is of great importance during metastasis since it plays a role in many steps. In Figure 1g, we could identify a lump of PTC metastasis invading a lymph node. Half of it was Galectin-3 strongly positive. We hypothesize that the half with stronger Galectin-3 expression was more aggressive compared to the other half since it was located heading the lymph nodes. This observation gave us a clue to link Galectin-3 with tumor migration and invasion. Together with the phenomenon of the higher expression of Galectin-3 in the frontier of the metastases, our primary assumption was that Galectin-3 might be involved in the process of PTC migration and invasion.

We next provided evidence that knocking down Galectin-3 reducing the migration and invasion of thyroid cancer cells. This result was in accordance with previous studies in other cancers [33–35]. The regulation function of Galectin-3 on cell migration was closely related to its ligands [36–38]. That's why Low-molecular Citrus Pectin (LCP) was used as a second method to inhibit the function of Galectin-3. Modified citrus pectin was first reported to decrease tumor metastases in lungs [39, 40]. Later its role in inhibiting melanoma cells adhesion to laminin was revealed [41]. In papillary thyroid cancer cells, citrus pectin also displayed a dose-dependent inhibition to the binding of Galectin-3 to human umbilical vein endothelial cells [42].

The impact of Galectin-3 inhibition on cell proliferation varied across studies [34, 35, 43]. In our study, knocking down Galectin-3 in B-cpap cells resulted in slightly decreased cell proliferation, while no differences were observed in 8305c cells. This difference may be due to different properties of the cell lines. An innovative finding of our study was that Galectin-3 knockdown significantly decreased the sphere formation ability of 8305c cells. And a reduction in the expression of Oct3/4, a marker of pluripotency and self-renewal, was also observed in 8305c Galectin-3 knockdown cells compared to control cells. Studies in thyroid and fibroblast have reported that overexpression of Galectin-3 caused malignant transformation [15, 21, 26]. However, Ilmer M. et al reported an association between loss of Galectin-3 and epithelial-to-mesenchymal transition (EMT) [44]. Altogether, Galectin-3 plays a certain role in cell motility, but its role in cancer cell proliferation and EMT needs to be further studied.

In addition, we also explored the mechanisms by which Galectin-3 regulated tumor cell migration. MAPK, Wnt/β-catenin and Src-FAK signaling pathways were first considered. The close relationship between Galectin-3 and β-catenin has already been verified in multiple studies [9, 10, 45]. After knocking down Galectin-3, we found the expression of β-catenin was suppressed, and its binding partner GSK3β was also deactivated through phosphorylation. The Wnt/β-catenin pathway is involved in the regulation of cell migration through modulating matrix metalloproteinases [46]. The suppressed expression of phosphorylated ERK (p-p44/42 MAPK) following Galectin-3 knockdown observed in our studies was in agreement with previous studies [45]. MAPK signaling modulates the proliferation, migration, and invasion of tumor cells [47, 48]. An ERK1/2–MEK1 module targets specific substrates involved in cell migration [49]. The ERK signaling pathway also controls the expression of matrix metalloproteinases and regulates the degradation of extracellular matrix proteins, which promotes cell migration and invasion as a result [48]. Src is highly activated in various human cancers [50]. Src regulates the disruption of adheren junctions and focal-adhesion turnover, which are required for cellular motility. FAK is one of the Src substrates and is related to integrin-mediated cellular motility, adhesion and invasion [51]. In the results of our study, phosphorylated Src and phosphorylated FAK (Tyr^397^) were suppressed after Galectin-3 knockdown. Caveolin-1 (Cav-1) is a multifunctional protein with multiple binding partners that is associated with cell surface caveolae and the regulation of lipid raft domains [52]. Cav1, together with the Mgat5/Galectin-3 lattice, was reported to regulate FAK dynamics in focal adhesions and favors focal adhesion disassembly and cell migration [53]. Jay Shankar et al reported the co-expression of Cav-1 and Galectin-3, which was exactly the same as our Immunofluorescence results [21]. In the same research, they also reported that Galectin-3 and Cav-1 were required for RhoA GTPase activation and knocking down of either Galectin-3 or Cav-1 significantly reduced the migration of differentiated thyroid cancer cells. Rho proteins play a critical role in regulating the actin cytoskeleton, the integrity of cell–extracellular matrix and cell–cell adhesions [54, 55]. The relationship between Galectin-3 and RhoA GTPase has also been reported by Nobuko Serizawa et al [22]. In addition to the RhoA GTPase, RhoC GTPase, whose role appears to weaken adherens junctions, was also investigated in our study [55]. Inhibition of Galectin-3 decreased the activation of RhoA GTPase as well as RhoC GTPase.

It has been proven that stressed microenvironments like hypoxia would cause a selection and malignant cells with more aggressive traits related to migration and metastasis may eventually become the dominant cell groups [23]. Galectin-3 was believed to modulate the adaptive strategies of cancer cells in stressed tumor microenvironments [25]. Galectin-3 overexpression was observed in hypoxic fields of cancer tissues [56, 57]. Under hypoxic conditions, the increase of Galectin-3 expression might act either as inducers of cell death or a pro-survival response triggered by stressed microenvironments [57]. Up-regulation of Galectin-3 in a hypoxic microenvironment relies on transcription factors such as HIF-1α and NF-κB [57, 58].

In our following experiments, B-cpap cells migrated faster in hypoxic microenvironments. Our hypothesis was that Galectin-3 induced this adaptive increase of cell migration. The wound healing results of B-cpap transfected cells proved that knocking down Galectin-3 attenuated the migration of PTC cells in hypoxic microenvironments but was not able to completely offset it. Thus, we supposed that there must exist some other factors that also modulate the migration of tumor cells in hypoxic microenvironments. The phenomenon of the deactivation of Src signaling indicated that Src signaling might be responsible for the Galectin-3 related increased migration under hypoxic conditions. The unchanged level of phosphorylated FAK supports the idea that FAK is not necessarily required for the increased motility that is induced by Src activation [50]. To the best of our knowledge, our study was the first one to relate Src signaling with cell migration regulated by Galectin-3 in hypoxic microenvironments. Taken together, up-regulation of Galectin-3 contributed to the increased tumor cell migration in response to hypoxic microenvironments.

In conclusion, our study showed that high expression of Galectin-3 was common in thyroid cancer metastases. Its function in regulating the migration of thyroid cancer cells was confirmed, probably through multiple signaling pathways. In a condition of lacking oxygen, Galectin-3 promotes cell motility by activating Src signaling. Studies using Galectin-3 targeted treatments for invasive papillary cancers have achieved some positive results [59]. While more researches are needed before this treatment could be applied in clinical practice. Understanding the mechanisms of how Galectin-3 promotes cancer cell motility may help to fulfill this goal.

## MATERIALS AND METHODS

### Sample collection and immunohistochemistry

Twenty patients accepted surgical excision as their primary treatment at Shanghai Zhongshan Hospital from April 2015 to August 2015. Among these patients, 19 were diagnosed with papillary thyroid cancers, including 9 patients with clinical positive lymph nodes. The rest one patient was diagnosed with anaplastic thyroid cancer. Pathological diagnoses were verified by qualified pathologist. Paraffin blocks containing representative cancer tissue were selected for immunohistochemical studies. The procedure for immunohistochemical analysis was performed as described previously [60]. Surgical specimens were stained with Galectin-3 antibody at a dilution of 1:100. Tissues of papillary thyroid cancer and adjacent noncancerous thyroid tissues were collected from another 14 PTC patients who also underwent surgeries in Zhongshan hospital. All tissues were kept in liquid nitrogen immediately after excision and were stored at -80°C until analysis. This study was approved by the ethics committee of Shanghai Zhongshan Hospital affiliated to Fudan University. All patients provided informed consent.

### Cell culture and transfection

Human papillary thyroid cancer cell line B-cpap and anaplastic thyroid cancer cell line 8305c were bought from Cell Bank affiliated to China Academy of Science. B-cpap cells were cultured in RPMI1640 (Gibco 12633-020) supplemented with 10% fetal bovine serum (Sigma 12003C), and 8305c cells were cultured in DMEM (Gibco 11965–09) supplemented with 10% fetal bovine serum (Sigma 12003C). 1% Penicillin and streptomycin (Gibco 15240-062) were added. All cells were cultured in a stable humidified environment with 5% CO2 at 37°C. The shRNA sequences included: Gal-3-shRNA (5’-CCCACGCTTCAATGAGAAC-5’) and Control-shRNA (5’-GCAAACAGAATTGCTTTAG-3’).

### Galectin-3 antagonism

Low-molecular citrus pectin (LCP) was dissolved in culture medium with a finial concentration of 0.5 mg/ml, 1 mg/ml, 2 mg/ml and 5 mg/ml accordingly. In the Wound healing assays, the concentration used was 2 mg/ml. LCP was obtained from Centrax.

### Hypoxia and inhibition of HIF-1a with MEO2

CoCl_2_ (100 μM) was added into the culture medium to mimic a hypoxic microenvironment. For HIF-1α inhibition studies, B-cpap cells were exposed in CoCl_2_-induced hypoxia and FBS deprivation microenvironment for 20 h. 10 μM MeOE2 (SELLECK) or DMSO, as vehicle, was added.

### RT-PCR

Total RNA was extracted using TRIzol solution (Invitrogen), and reverse transcription PCR was performed using an Advantage RT-for-PCR kit (Takara) according to the manufacturers’ instructions. The RT-PCR reactions were carried out with the following primers: human LGALS3 (sense-5’-TAA TAA CTG GGG AAG GGA AG-3’, anti-sense-5’- AGC ACT GGT GAG GTC TAT GT-3’), β-actin (sense-5’- CAT CCA CGA AAC TAC CTT CAA CT CC-3’, anti-sense-5’- GAG CCG CCG ATC CAC ACG-3’), and HIF-1α (sense-5’- ACG TGT TAT CTG TCG CTT TG-3’, anti-sense-5’- TAG GTT TCT GCT GCC TTG TAT-3’). All reactions were performed in Mastercycler personal (Eppendorf).

### Rho-activation assay

B-cpap cells transfected with control- and Gal-3-shRNA were lyzed and the supernatant was collected for the pull-down assay using a Rho activation assay kit. In brief, the cell lysates were mixed with glutathione-agarose beads bound with Rhotekin, containing a GTP-Rho binding domain. After incubation, the agarose beads were washed and collected by centrifugation, then resuspended in the sample buffer containing 1M dithiothreitol. The active GTP-Rho was released and denatured by boiling. The supernatant was collected and separated by SDS-polyacrylamide gel. The GTP-bound RhoA and RhoC were detected by the antibodies from CST.

### Immunofluorescence

Frozen sections of the surgical specimens were prepared by the department of pathology, Zhongshan hospital of Fudan University. The patient involved provided an informed consent. The sections were fixed with acetone for 20 min at room temperature, washed three times with PBS, and blocked with a PBS-based solution containing 5% normal serum. Then they were incubated overnight at 4°C with mouse anti-Cav-1 (1:200) and rabbit anti-Galectin-3 (1:250) or rabbit anti-GLUT1 (1:200) and mouse anti-Galectin-3 (1:100). After being washed three times with PBS, the sections were incubated with donkey anti-mouse Alexa Flour 488 IgG (1:800) and donkey anti-rabbit Alexa Fluor 594 IgG (1:800), or donkey anti-rabbit Alexa Fluor 488 IgG (1:800) and donkey anti-mouse Alexa Fluor 594 IgG (1:800) for double immunofluorescence staining. Nuclei were counterstained with Hoechst 33258 (10 μg/ml). Immunofluorescence images were collected on a Leica TCS SP5 confocal microscope and analyzed using LAS AF software.

### Western blot analysis

The procedure for Western blotting analysis was performed as described previously [65]. The dilution ratios of the primary antibodies were as follows: Galecin-3(mouse), 1: 1000; p44/42 MAPK, 1:1000; p-p44/42 MAPK, 1:2000; Src, 1:1000; p-Src, 1:1000; FAK, 1:1000; p-FAK-Tyr^397^, 1:1000; β-catenin, 1:1000; GSK-3β, 1:1000; p-GSK-3β, 1:1000; Oct3/4 1:500; RhoA 1:1000; RhoC, 1:1000; Cav-1, 1:500 and HIF-1α, 1:250. β-actin (1:1000) was used as a loading control. The quantification of the band density was analyzed by Gel Image System (Tanon).

### Cell proliferation assay

Cell proliferation was detected using Cell Counting Kit8 (Dojindo) according to the manufacturer's instructions. Briefly, 8305c and B-cpap cells were plated in 96-well plates at a density of 3,000 cells per replicate. Reagent was added at the same time for the following days. After incubation for 2 hours, the absorbance of each well was determined at 450 nm using a Gen5 system (BioTek).

### Wound healing assay

For the wound healing assays, cells were cultured in 6-well plates. When 100% confluence was reached, the cell monolayers were wounded with a pipette tip. Then the cells were washed by PBS and photographed. Five fields of view were chosen in each plate, and all assays were performed in duplicate.

### Transwell assay

Matrigel was diluted in serum-free culture medium at a ratio of 1:8 and tiled on the upper chambers of a 24-well transwell plate (8 μm; Corning Costar Co., Cambridge, MA). Once the Matrigel solidified, the cells were trypsinized and suspended in serum-free medium to a final concentration of 5×10^4^/ml. 200 μl of cell suspension was added into the upper chamber, and 600 μl of culture medium with 10% FBS was added into the lower chamber. After 20 h, the cells on the upper side of the membrane were removed with cotton swabs, and the cells that migrated to the lower surface were fixed with methanol for 30 min and stained with 0.1% crystal violet for 20 min. The cells were counted in five fields of view in duplicate chambers.

### Sphere formation assay

The sphere formation assays were carried out in 96-well plates. Briefly, an average of 300 cells were plated in each well. The culture medium was Dulbecco's modified Eagle's and F12 media supplemented with B27 (Invitrogen), 2 μg/ml heparin (Sigma), 20 ng/ml EGF (Chemicon) and 20 ng/ml FGF-2 (Chemicon). The total number of formed spheres and their average diameters were counted after 14 days.

### Reagents and antibodies

The mouse anti-Galectin-3 (BD556904) antibody was obtained from BD Biosciences. The rabbit anti-Galectin-3 (Ab31707) antibody was obtained from abcam. The rabbit anti-RhoA (CST2117), rabbit anti-RhoC (CST3430), rabbit anti-FAK (CST3285), rabbit anti-pFAK-Tyr^397^ (CST8556), rabbit anti-p44/42 MAPK (CST4695), rabbit anti-p-p44/42 MAPK Thr^202^/Tyr^204^ (CST4370), rabbit anti-β-catenin (CST8480), rabbit anti-GSK-3β (CST9315), rabbit anti-p-GSK-3β Ser^9^ (CST9336), rabbit anti- Src (CST2108) and rabbit anti-p-SrcFamily Tyr^416^ antibodies (CST2101) were obtained from Cell Signaling Technology. The rabbit anti-Oct3/4 (Sc9801), mouse anti-Cav-1 (sc53564) antibodies were obtained from Santa Cruz Biotechnology. The rabbit anti-GLUT1 (PA5-16793) antibody was obtained from Thermo Fisher. Rho activation assay kit (17-294) and PP2 were obtained from Merck Millipore.

### Statistical Analysis

Statistical analysis was performed using SPSS 21. Comparisons of categorical data were carried out by unpaired two-tailed Student's t test. Data are presented as the mean ± S.D. *p* values < 0.05 were considered statistically significant.
